# Damage assessment of suspension footbridge using vibration measurement data combined with a hybrid bee-genetic algorithm

**DOI:** 10.1038/s41598-022-24445-6

**Published:** 2022-11-22

**Authors:** Lan Ngoc-Nguyen, Hoa Ngoc-Tran, Samir Khatir, Thang Le-Xuan, Quyet Huu-Nguyen, G. De Roeck, Thanh Bui-Tien, Magd Abdel Wahab

**Affiliations:** 1 Laboratory Soete, Department of Electrical Energy, Metals, Mechanical Constructions, and Systems, Faculty of Engineering and Architecture, Ghent University, 9000 Gent, Belgium; 2 Department of Bridge and Tunnel Engineering, Faculty of Civil Engineering, University of Transport and Communications, Hanoi, Vietnam; 3 Faculty of Civil Engineering, Ho Chi Minh City Open University, Ho Chi Minh City, Viet Nam; 4 Department of Civil Engineering, KU Leuven, 3001 Leuven, Belgium; 5 Faculty of Mechanical-Electrical and Computer Engineering, School of Engineering and Technology, Van Lang University, Ho Chi Minh City, Vietnam

**Keywords:** Civil engineering, Computational science

## Abstract

Optimization algorithms (OAs) are a vital tool to deal with complex problems, and the improvement of OA is inseparable from practical strategies and mechanisms. Among the OAs, Bee Algorithm (BA) is an intelligent algorithm with a simple mechanism and easy implementation, in which effectiveness has been proven when handling optimization problems. Nevertheless, BA still has some fundamental drawbacks, which can hinder its effectiveness and accuracy. Therefore, this paper proposes a novel approach to tackle the shortcomings of BA by combining it with Genetic Algorithm (GA). The main intention is to combine the strengths of both optimization techniques, which are the exploitative search ability of BA and the robustness with the crossover and mutation capacity of GA. An investigation of a real-life suspension footbridge is considered to validate the effectiveness of the proposed method. A baseline Finite Element model of the bridge is constructed based on vibration measurement data and model updating, which is used to generate different hypothetical damage scenarios. The proposed HBGA is tested against BA, GA, and PSO to showcase its effectiveness in detecting damage for each scenario. The results show that the proposed algorithm is effective in dealing with the damage assessment problems of SHM.

## Introduction

Civil engineering structures, including bridges, highways, and dams, experience deterioration during their service life. Structures deteriorate due to various factors, including fatigue caused by overload traffic or loads of extreme events such as earthquakes, floods, or accidental loads. These damages threaten the safety of the structures in the long run, which may consequently lead to catastrophic failure or collapse. Structural damage detection at the earliest possible stage has become the core research in the field of civil engineering for many years^1^. Among the current non-destructive evaluation techniques, Structural Health Monitoring (SHM) has emerged to be an indispensable system for the construction and maintenance of modern lifeline infrastructures^2^. SHM is the process of structural inspection and evaluation over time using a wide range of sensors to collect different information that may affect the working condition of the structure, such as displacement, temperature, wind level, etc. of the structure. Based on the health evaluation of the structure, an early repair and strengthening plan can be made to maintain the safety and integrity of the structure, which would help to reduce the maintenance cost significantly. Throughout the years, the task of improving the damage detection capabilities of SHM systems has captured the attention of many researchers all over the world^3–10^. Although many improvements and progress have been made, the “ultimate” damage detection tool, which is the one to solve them all, is still yet to be found. This is one of the reasons why more and more efforts are being spent to augment the efficacy of SHM.

Recently, the emergence of OAs has immensely unlocked the potential of many problem solvers in dealing with complex issues^11–13^. The application of OAs has also proven to be extremely compelling in enhancing the effectiveness of SHM systems^14–17^. The OAs help to minimize the difference between calculated and measured results. Then, the calibrated model possibly predicts the behaviour of the considered structures accurately. In recent decades, many researchers have successfully applied OAs to improve the efficacy of SHM tools. For instance, Nguyen-Ngoc et al.^18^ proposed using Particle Swarm Optimization (PSO) algorithm coupled with an Artificial Neural Network (ANN) for damage detection, in which the proposed method was accurately superior compared to traditional PSO and ANN in detecting damages of a bridge. A novel hybrid algorithm combined with experimental data from wireless triaxial sensors was proposed by Tran et al.^19^ and applied for model updating of a large-scale truss bridge. In the work of^20^, two recently proposed algorithms, namely Salp Swarm Optimizer (SSA) and Atom Search Optimization (ASO) were used to identify damages in a 37-bar planar truss, a 52-bar planar truss, and a 52-bar space truss.

Bee Algorithm (BA) was first proposed in 2006 by Pham et al.^21^, which mimicked the food-foraging process of a honeybee colony. Since then, this algorithm has become one of the most widely used swarm-based algorithms in solving optimization problems. For example, Kavoussi et al.^22^ employed BA to eliminate low-order harmonics in a cascaded multilevel inverter. The obtained results outperformed GA in terms of accuracy. BA was also used to optimize the wireless sensor network coverage in the work of^23^. Niknam et al.^24^ proposed an improved BA to optimize the placement of renewable electricity generators. Nevertheless, BA still has main drawbacks, such as easily being trapped in local minima, slow convergence speed, and depending mainly on the quality of the initial populations. These issues may reduce its effectiveness, especially in solving the optimization of complex structures. Hence, in this work, to avoid these problems, we propose a novel hybrid algorithm combining BA with GA to deal with the damage detection problem of a suspension bridge. The working principle of the proposed approach is that BA with global search techniques is used to generate initial populations with high quality; then GA with crossover and mutation operator is used to improve the quality of the populations after each iteration. According to the No Free Lunch Theorem, no algorithm can solve all the optimization problems. For the other metaheuristic algorithms, many researchers have tried to improve them such as in the works of^11,14,24,31^. Therefore, in this work, we aim to improve the BA algorithm to enrich the algorithm systems to solve optimization problems. For a better illustration, the main contributions of this work are depicted as follows:

- A hybrid algorithm combining BA and GA is proposed to enhance the effectiveness of traditional BA and GA.
- The effectiveness and correctness of the proposed method are proved by detecting damages for a real-life suspension bridge, in which both single and multiple damages are considered.
- A comparison between the proposed method and numerous other algorithms, including traditional GA and BA is conducted.

This paper is split into four main sections. Apart from the introduction part, the methodology of BA, GA, and the proposed method is introduced in “Methodology”. The case study of the Na Xa suspension footbridge is presented in “Application of HBGA to damage detection of Na Xa suspension footbridge”, along with the evaluation of the effectiveness of the proposed approach, respectively. Finally, the main conclusions are drawn.

## Methodology

### Bee algorithm

Since its first introduction in 2006 by Pham et al.^21^, BA has become one of the most widely used swarm-based algorithms in solving optimization problems. The bee algorithm is inspired by the food-foraging process of a honeybee colony. During the harvesting season, a colony will try to maximize its food-searching capacity by sending several scout bees to look for food. The scout bees search for food randomly to identify flower patches that contain plenties of nectar. When they return to the hive, the nectar qualities are assessed. If the nectar’s quality is judged to be above the standard threshold, the scout bee will perform a ritual called “waggle dance”^25^. The purpose of this ritual is to notify the colony of different information regarding the high-quality nectar it found, including the location of the food source, its distance from the colony, and its rating of the nectar quality. Based on that information, the required number of bees to forage nectar at the food source could be estimated and dispatched effectively. This process helps to optimize the food-foraging process of the bee colony and ensure its survivability.

In the Bee algorithm, the bee population is divided into two main groups: “scout” bees and “elite” bees. “Scout” bees are tasked to explore randomly in the solution space to look for possible high-fitness solutions. Once the good solutions are identified, the “elite” bees are then employed to perform a local search within these solutions to look for further possible fitness improvement. The number of “elite” bees selected for a particular neighborhood depends on the ranking of the fitness of visited solutions, in which the selected process imitates the “waggle dance” of bees in real life. From there, the new solutions which have similar values to the current best finds are created and assessed. The “waggle dance” of the bees is formulated as shown in Eq. (1) below.

1 $$y=x+k$$

where $y$ is the new position of the bee and $x$ is the old position of the bee, respectively. $k$ is a random parameter with values between [-*r*,*r*], in which *r* is the radius of the bee’s patches.

### Genetic algorithm

Genetic Algorithm (GA) is a metaheuristic search algorithm that was first proposed in 1973 by John Holland^26^. The creation of GA is considered the founding pillar of the instrumental rise of the so-called “evolutionary algorithms (EA). For more than 40 years, GA has remained to be one of the most widely used OAs in solving optimization problems, especially in civil engineering^27–31^. The algorithm is inspired by Darwin’s theory of natural evolution^33^, which mimics the natural selection process of biological genetics, where only the fittest individuals are selected for reproduction to evolve better progenies for the next generation.

GA consists of three main operators, which are used to produce the next generation from the current population: selection, crossover, and mutation. *Selection* helps to determine the input of the reproduction process by selecting the individuals so-called “parents” arbitrarily. This operator also indicates the convergence rate of GA in solving optimization problems. Some of the well-known selection techniques are tournament, Boltzmann, roulette wheel, etc.^34^. For this research, the roulette wheel is selected for the selection process of GA since it can prevent too quick convergence from happening, which may cause the missing of minima points. *Crossover* operator is used to combine the genetic information of two or more parents to form offspring. This can be done by replacing the genes of one pair of the parent with the respective genes of the other. The third operator-*mutation* is introduced in GA to generate the genetic diversity from the parent's population to the subsequent offspring. The mutation is used to avoid the trapping of local minima in the optimization process by preventing the generation of identical offspring, which may cause disruption in the convergence process to achieve the global optimum of the problem. In GA, the mutation is formulated as follows:

2 $$y=x+\sigma *random$$

3 $$\sigma = \alpha *\frac{Var\_\mathrm{max}- Var\_min}{2}$$

where $x$ and $y$ is the old and new position of the search, respectively; *random* is a random parameter with the values between [-1,1] and *α* is the parameter encoding the maximum width of the mutation events.

### Hybrid bee-genetic algorithm (HBGA)

As demonstrated above, while BA is an advanced optimization, it still possesses its shortcomings. In BA, the search for the food source is dependent entirely on the capability and the numbers of the “scout” bees. If the “scout” bees indicate that the searched space is prone to be the best (while in fact it might not be!), it will send the information back to the colony where “elite” bees will be dispatched to search for the food in that region, which leads to a situation where they will be trapped in local minima. Moreover, since the number of non-selected “elite” bees dispatched to this trapped region is significantly high, only the selected bees are chosen to search for food preliminary evaluation information. This is a waste since those non-selected bees can still search for the global optimum. Meanwhile, GA is known for dealing with local minima problems effectively with the help of crossover and mutation operators. However, it would not be able to store the preceding data since it was mutated and crossover in the previous generation. That is why the Hybrid Bee-Genetic Algorithm (HBGA) is proposed. For the global search, instead of searching arbitrarily, non-selected bees will be cross-over and mutated by a cross factor and mutation factor. The results will be synthesized to select the best possible outcomes. The proposed hybrid algorithm shall help the search to avoid premature convergence due to being trapped in local minima whilst minimizing the chance of missing out the global optimum, as well as improving the convergent rate of the search algorithm. The process of HBGA is indicated as follows:

**Step 1:** Creating the initialization of the bee group:

4 $${X}_{sb}\left(0\right)=\left[\begin{array}{c}\begin{array}{ccc}{x}_{1}^{1}& {x}_{2}^{1}& \begin{array}{cc}\dots & {x}_{m}^{1}\end{array}\end{array}\\ \begin{array}{ccc}{x}_{1}^{2}& {x}_{2}^{2}& \begin{array}{cc}\dots & {x}_{m}^{2}\end{array}\end{array}\\ \begin{array}{c}\begin{array}{ccc}\dots & \dots & \begin{array}{cc}\dots & \dots \end{array}\end{array}\\ \begin{array}{ccc}{x}_{1}^{{n}_{sb}}& {x}_{1}^{{n}_{sb}}& \begin{array}{cc}\dots & {x}_{m}^{{n}_{sb}}\end{array}\end{array}\end{array}\end{array}\right]$$

where ${n}_{sb}$ is the number of “scout” bees, *m* is the search dimension.

**Step 2:** Determination of the Fitness Evaluation:

5 $${F}_{sb}(0)=\left[\begin{array}{c}{f}^{1}\\ {f}^{2}\\ \begin{array}{c}\vdots \\ {f}^{{n}_{sb}}\end{array}\end{array}\right]$$

**Step 3:** Sorting of bees and updating the solution for ${X}_{sb}\left(0\right)$ sorted.

**Step 4:** Arranging the bees into two groups: non-selected Site and selected Site.

- Group 1: Selected Site (*n*_*s*_)
  The number of “scout” bees *n*_*s*_ in the non-selected site is calculated as:
  6 $${n}_{s}={n}_{sb}-{n}_{ns}$$
  where ${n}_{sb}$ indicates the total number of “scout” bees, ${n}_{ns}$ indicates the number of bees of the non-selected site.
  For the Elite Site (es):
  The number of bees in the Elite Site (*n*_*es*_) is selected as:
  7 $${n}_{es}=round({\upgamma }_{es}*\frac{{n}_{ns}}{2})$$
  where ${\upgamma }_{es}=0.4$ is the selection factor for the Elite Site group.
  Calculating the new position in ${n}_{es}$ , we obtain:
  8 $${X}_{es}\left(it\right)=\left[\begin{array}{c}\begin{array}{ccc}{x}_{1}^{1}& {x}_{2}^{1}& \begin{array}{cc}\dots & {x}_{m}^{1}\end{array}\end{array}\\ \begin{array}{ccc}{x}_{1}^{2}& {x}_{2}^{2}& \begin{array}{cc}\dots & {x}_{m}^{2}\end{array}\end{array}\\ \begin{array}{c}\begin{array}{ccc}\dots & \dots & \begin{array}{cc}\dots & \dots \end{array}\end{array}\\ \begin{array}{ccc}{x}_{1}^{{n}_{es}}& {x}_{1}^{{n}_{es}}& \begin{array}{cc}\dots & {x}_{m}^{{n}_{es}}\end{array}\end{array}\end{array}\end{array}\right]$$
  Recalculating the result of these elite bees to obtain the new elite site:
  9 $$F_{es} \left( {it} \right) = \left[ {\begin{array}{*{20}c} {f^{1} } \\ {\begin{array}{*{20}c} {f^{2} } \\ \vdots \\ \end{array} } \\ {f^{{n_{es} }} } \\ \end{array} } \right]$$
  For the Other Site, which is the group of bees not selected by the “scout” bees, but belongs to the selected site, the total number of “scout” bees in this group is calculated as:
  10 $${n}_{os}=round(\left(1-{\upgamma }_{es}\right)*\frac{{\mathrm{n}}_{\mathrm{ns}}}{2})$$
  Calculating the new position in ${n}_{os}$ , we obtain:
  11 $${X}_{os}\left(it\right)=\left[\begin{array}{c}\begin{array}{ccc}{x}_{1}^{1}& {x}_{2}^{1}& \begin{array}{cc}\dots & {x}_{m}^{1}\end{array}\end{array}\\ \begin{array}{ccc}{x}_{1}^{2}& {x}_{2}^{2}& \begin{array}{cc}\dots & {x}_{m}^{2}\end{array}\end{array}\\ \begin{array}{c}\begin{array}{ccc}\dots & \dots & \begin{array}{cc}\dots & \dots \end{array}\end{array}\\ \begin{array}{ccc}{x}_{1}^{{n}_{os}}& {x}_{1}^{{n}_{os}}& \begin{array}{cc}\dots & {x}_{m}^{{n}_{os}}\end{array}\end{array}\end{array}\end{array}\right]$$
  Recalculating the result of these elite bees to obtain the new other site:
  12 $${F}_{os}\left(it\right)=\left[\begin{array}{c}{f}^{1}\\ \begin{array}{c}{f}^{2}\\ \vdots \end{array}\\ {f}^{{n}_{os}}\end{array}\right]$$
  The outcome for the selected site will be calculated based on Eq. (9) and (11) as:
  13 $${X}_{s}\left(it\right)=\left[\begin{array}{c}\begin{array}{ccc}{x}_{1}^{1}& {x}_{2}^{1}& \begin{array}{cc}\dots & {x}_{m}^{1}\end{array}\end{array}\\ \begin{array}{ccc}{x}_{1}^{2}& {x}_{2}^{2}& \begin{array}{cc}\dots & {x}_{m}^{2}\end{array}\end{array}\\ \begin{array}{c}\begin{array}{ccc}\dots & \dots & \begin{array}{cc}\dots & \dots \end{array}\end{array}\\ \begin{array}{ccc}{x}_{1}^{{n}_{s}}& {x}_{1}^{{n}_{s}}& \begin{array}{cc}\dots & {x}_{m}^{{n}_{s}}\end{array}\end{array}\end{array}\end{array}\right]$$
  where ${X}_{s}$ is the updated position of the selected site.
  The fitness function of the selected site is calculated as Eq. (14) below:
  14 $${F}_{s}\left(it\right)=\left[\begin{array}{c}{F}_{es}\left(it\right)\\ {F}_{os}\left(it\right)\end{array}\right]$$
- Group 2: Non-selected Site (${n}_{ns}$):

The total number “scout” bees in the non-selected site ${n}_{ns}$ is calculated as:

15 $${n}_{ns}=0.5* {n}_{sb}$$

#### Crossover

For crossover, the total number of bees for crossover ($${n}_{c})$$ is calculated based on a random number of parents are chosen for crossover with the crossover factor $${\upgamma }_{c}$$ =0.8:

16 $${n}_{c}=2*round({\upgamma }_{c}*\frac{{\mathrm{n}}_{\mathrm{ns}}}{2})$$

The new position of the bees is updated with the crossover number by:

17 $$\begin{gathered} y_{1} = \left[ {\alpha *x_{1} + \left( {1 - \alpha } \right)*x_{2} } \right]*w \hfill \\ y_{2} = \left[ {\alpha *x_{2} + \left( {1 - \alpha } \right)*x_{1} } \right]*w \hfill \\ \end{gathered}$$

where: ${y}_{1},{y}_{2}$ are the offspring obtained by using crossover and ${x}_{1},$ ${x}_{2}$ are the parent bees respectively, and $w$=0.99 is the added weight which is calculated as:

18 $$w={w}_{\mathit{max}}-\frac{{w}_{max}-{w}_{min}}{T}it$$

With $it$ and $T$ are the current and maximum iteration, respectively.

The matrix of the new bee position created by using crossover is calculated as:

19 $$X_{c} \left( {it} \right) = \left[ {\begin{array}{*{20}c} {\begin{array}{*{20}c} {x_{1}^{1} } & {x_{2}^{1} } & {\begin{array}{*{20}c} \ldots & {x_{m}^{1} } \\ \end{array} } \\ \end{array} } \\ {\begin{array}{*{20}c} {x_{1}^{2} } & {x_{2}^{2} } & {\begin{array}{*{20}c} \ldots & {x_{m}^{2} } \\ \end{array} } \\ \end{array} } \\ {\begin{array}{*{20}c} {\begin{array}{*{20}c} \ldots & \ldots & {\begin{array}{*{20}c} \ldots & \ldots \\ \end{array} } \\ \end{array} } \\ {\begin{array}{*{20}c} {x_{1}^{{n_{c} }} } & {x_{1}^{{n_{c} }} } & {\begin{array}{*{20}c} \ldots & {x_{m}^{{n_{c} }} } \\ \end{array} } \\ \end{array} } \\ \end{array} } \\ \end{array} } \right]$$

Recalculating the value of the objective functions after crossover by the Eq. (20):

20 $${F}_{c}\left(it\right)=\left[\begin{array}{c}{f}^{1}\\ \begin{array}{c}{f}^{2}\\ \vdots \end{array}\\ {f}^{{n}_{c}}\end{array}\right]$$

#### Mutation

For mutation, the total number of bees for mutation ${n}_{mu}$ is calculated based on a random number of parents are chosen for mutation with the mutation factor ${\upgamma }_{mu}$ =0.1.

21 $${n}_{mu}=2*round({\upgamma }_{mu}*\frac{{\mathrm{n}}_{\mathrm{ns}}}{2})$$

The new population is updated with the mutation number as Eqs. (2) and (3) above.

22 $${X}_{mu}\left(it\right)=\left[\begin{array}{c}\begin{array}{ccc}{x}_{1}^{1}& {x}_{2}^{1}& \begin{array}{cc}\dots & {x}_{m}^{1}\end{array}\end{array}\\ \begin{array}{ccc}{x}_{1}^{2}& {x}_{2}^{2}& \begin{array}{cc}\dots & {x}_{m}^{2}\end{array}\end{array}\\ \begin{array}{c}\begin{array}{ccc}\dots & \dots & \begin{array}{cc}\dots & \dots \end{array}\end{array}\\ \begin{array}{ccc}{x}_{1}^{{n}_{mu}}& {x}_{1}^{{n}_{mu}}& \begin{array}{cc}\dots & {x}_{m}^{{n}_{mu}}\end{array}\end{array}\end{array}\end{array}\right]$$

Recalculating the value of the objective functions after mutation as:

23 $${F}_{mu}\left(it\right)=\left[\begin{array}{c}{f}^{1}\\ \begin{array}{c}{f}^{2}\\ \vdots \end{array}\\ {f}^{{n}_{mu}}\end{array}\right]$$

**Step 5:** Updating the new population.

The new population of bee is updated based on the previous population of selected site, crossover and mutation operators:

24 $${F}_{group}\left(it\right)=\left[\begin{array}{c}{F}_{s}\left(it\right)\\ {F}_{c}\left(it\right)\\ {F}_{mu}\left(it\right)\end{array}\right]$$

The best value from the new population is sorted from the updated new population ${F}_{group}\left(it\right)$:

25 $$F_{new} \left( {it} \right) = \left[ {\begin{array}{*{20}c} {f^{1} } \\ {\begin{array}{*{20}c} {f^{2} } \\ \vdots \\ \end{array} } \\ {f^{{n_{s} }} } \\ \end{array} } \right]$$

**Step 6:** Updating the value for the global best G_best_.

**Step 7:** Repeat **Step 4** until the criteria are satisfied or until the number of iterations is finished.

**Step 8:** The best solution is determined by:

26 $${\text{X }} = {\text{ G}}_{{{\text{best}}}}$$

**Figure 1:**
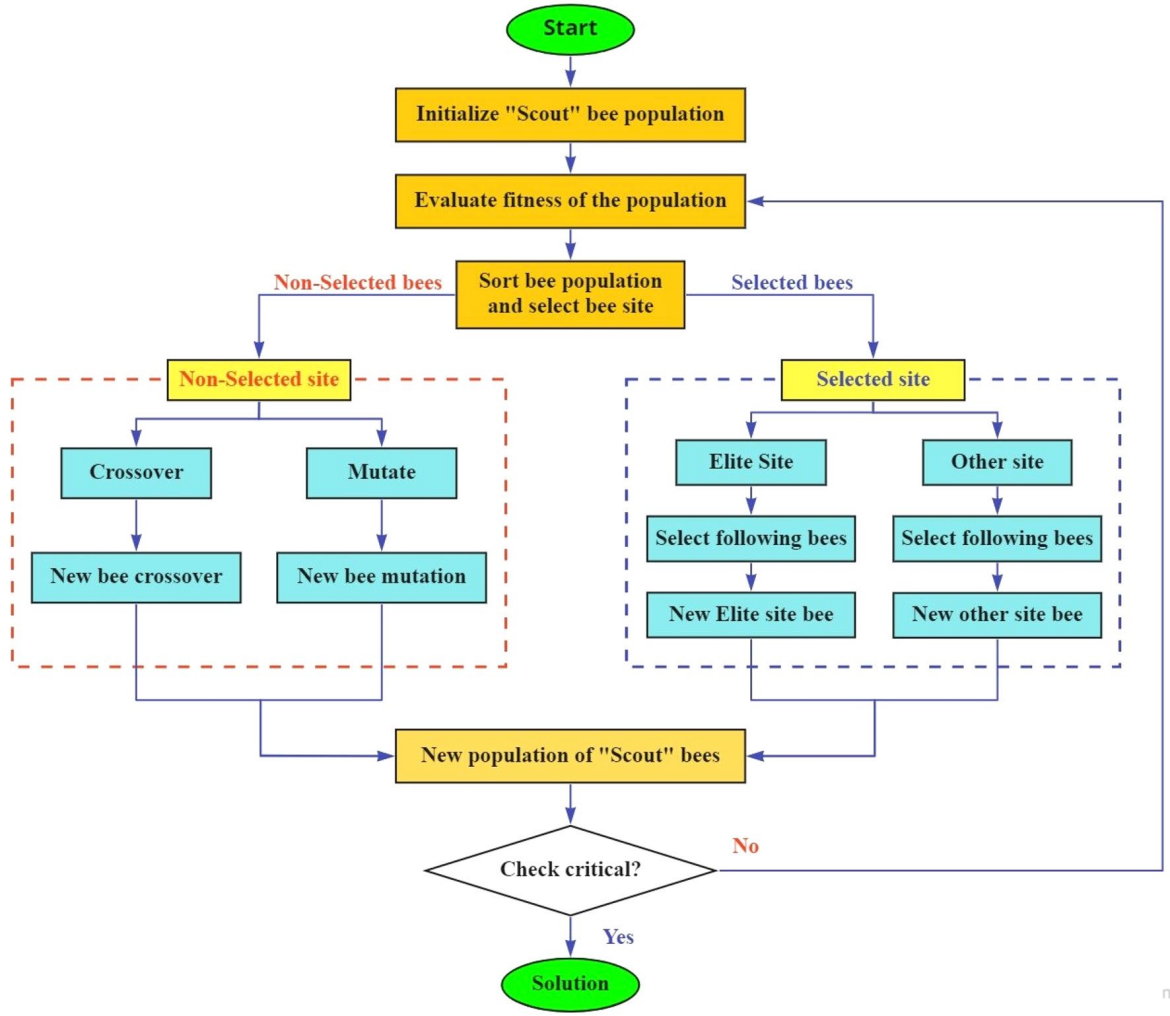
Flowchart of HBGA.

The flowchart of HBGA is shown in Fig. 1:

## Application of HBGA to damage detection of Na Xa suspension footbridge

### Introduction to Na Xa suspension footbridge

**Figure 2:**
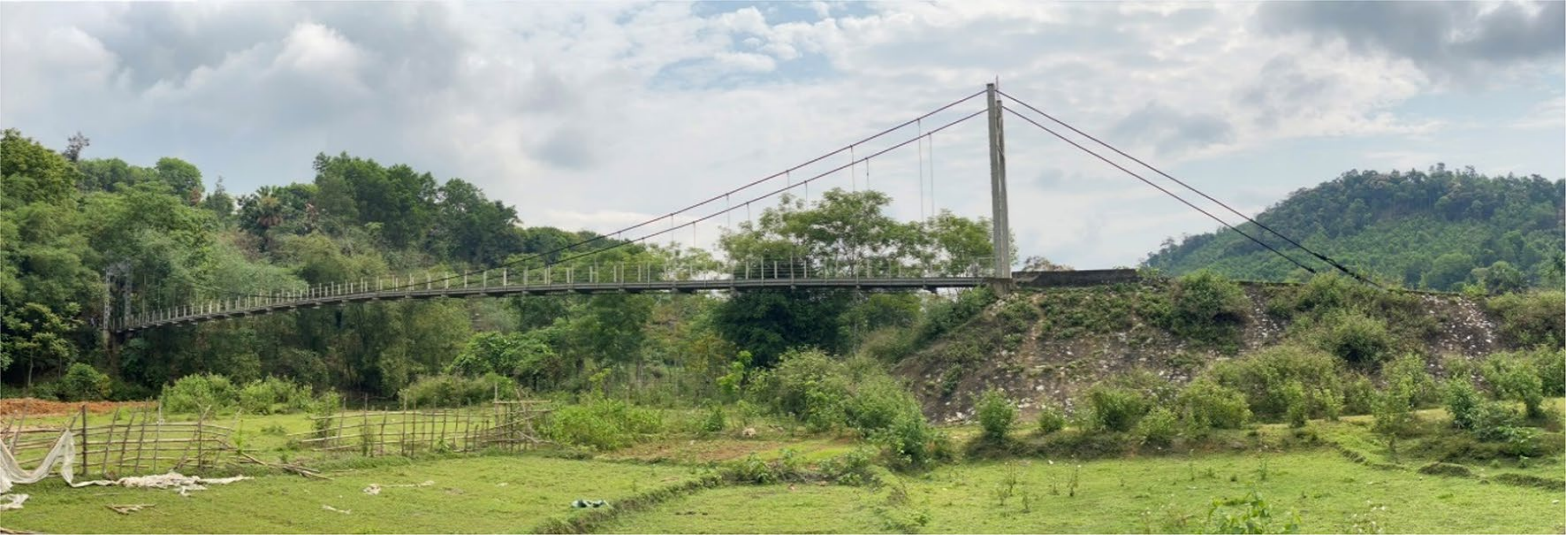
Na Xa suspension footbridge.

Located in the remote region of Nghe An Province, Vietnam, Na Xa bridge (Fig. 2) is one of the few suspension footbridges connecting the livelihoods of the tribal communes in the local area. The bridge has a total span length of 70.2 m with two high tensile main cables of a nominal diameter of 56 mm. Suspension cables are made of Φ20 mm steel cables. The bridge towers have a height of 8 m and consist of I-shape steels, which are fixed at 1 m deep into the concrete base at the tower leg. The abutments are made of M250-graded reinforced concrete. This bridge is a typical suspension bridge in the Mid-Northern mountainous area of Vietnam, where a lot of streams are presented.

**Figure 3:**
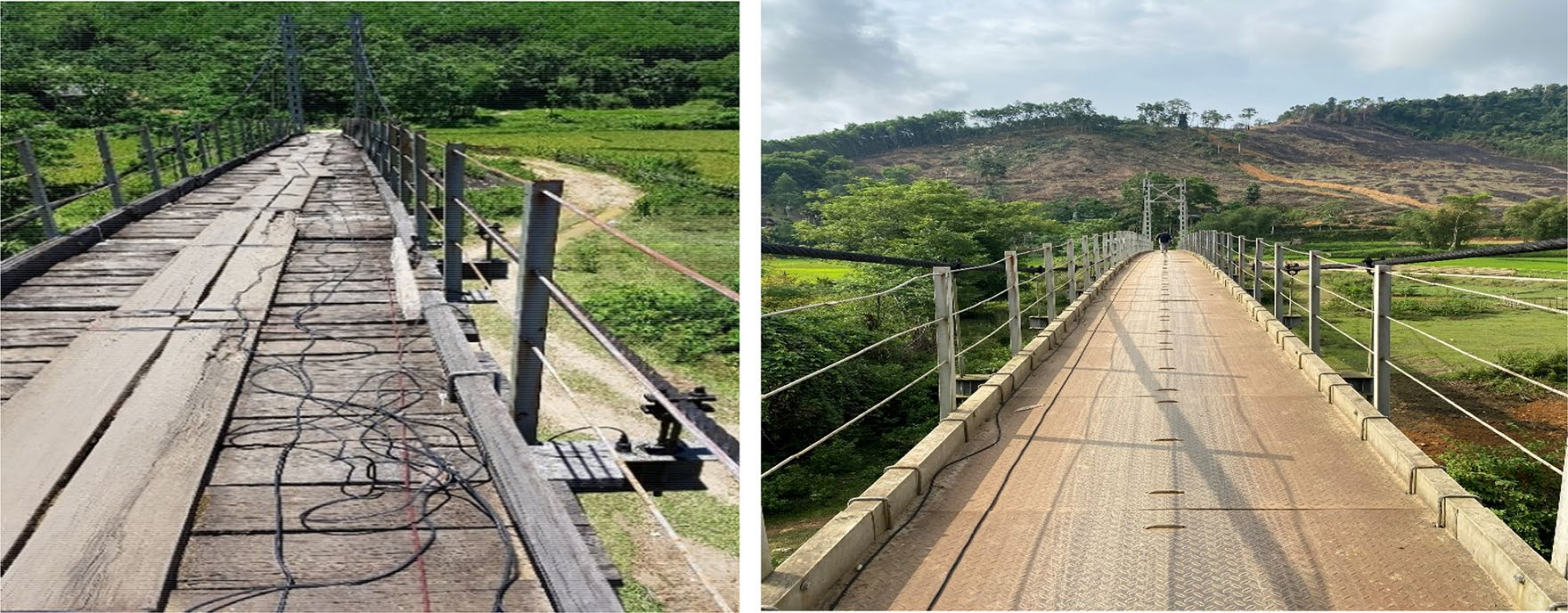
Na Xa bridge in 2016 (before retrofitting—left) and in 2022 (after retrofitting—right).

Prior to 2018, Na Xa bridge was under heavy deterioration, which caused uneasy vibration when pedestrians were crossing the bridge, hence threatening the integrity of the structure as well as the safety of the pedestrians. In 2018, the bridge was undergone retrofitting to repair different weak and damaged spots along the bridge, especially on the bridge’s deck. The bridge deck was replaced and strengthened from a wooden deck to 2 mm steel plate deck for the whole of the bridge. Additional steel bars with a diameter = 3 mm were added to reinforce the bridge deck (Fig. 3). All the cables were also re-tensioned according to the design standard of bridges. In April 2022, a vibration measurement campaign was conducted to assess the health of the structure after retrofitting.

### Finite element model

A Finite Element (FE) model of the bridge after retrofitting is constructed using the program ANSYS Parametric Design Language (APDL)^35^. Three different types of elements are applied to model the various components of the bridge. BEAM188 element is used to model the tower, the main beam, and the cross beam of the bridge. SHELL181 element is used to model the steel deck, and LINK180 element is used for the main cables and the suspension cables on the bridge. Non-structural components of the bridge, such as handrails, bolts, etc., are considered as additional masses on the bridge with the MASS21 element. A total of 330 nodes and 578 elements are assembling the model accordingly. The material properties of the bridge are assumed to be the same for all the bridge components with the following: Young’s modulus = 200 GPa*,* Poisson’s ratio = 0.3*,* Density = 7850 kg/m^3^.

#### Boundary conditions

The bottom of the tower legs are considered as fixed support. Fixed anchor supports are also considered at the anchorage points of the two extended main suspension cables and the additional suspension cables.

#### Connections

All connections are assumed to be rigid. Connections between the bridge deck and the main beam, the cross beam, and the main beam are realized using coupled degrees of freedom (CP) command in ANSYS.

**Figure 4:**
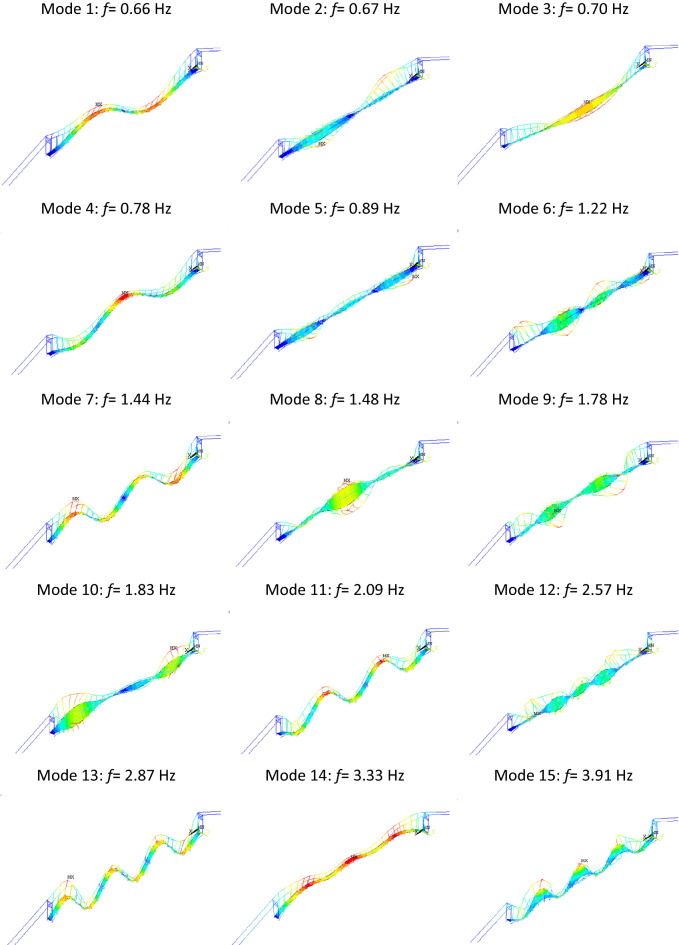
Obtained mode shapes and respected natural frequencies from the FE model.

After modal analysis, 15 natural frequencies and respected mode shapes are obtained, as shown below:

From Fig. 4, it can be seen that all of the identified mode shapes are global modes. However, the natural frequencies of the identified mode shapes are very close to each other. This is a noticeable issue that may make it more difficult to separate each mode, also during the analysis of the vibration measurement data.

### Collection and analysis of vibration measurement data

#### Collection of vibration measurement data

**Figure 5:**
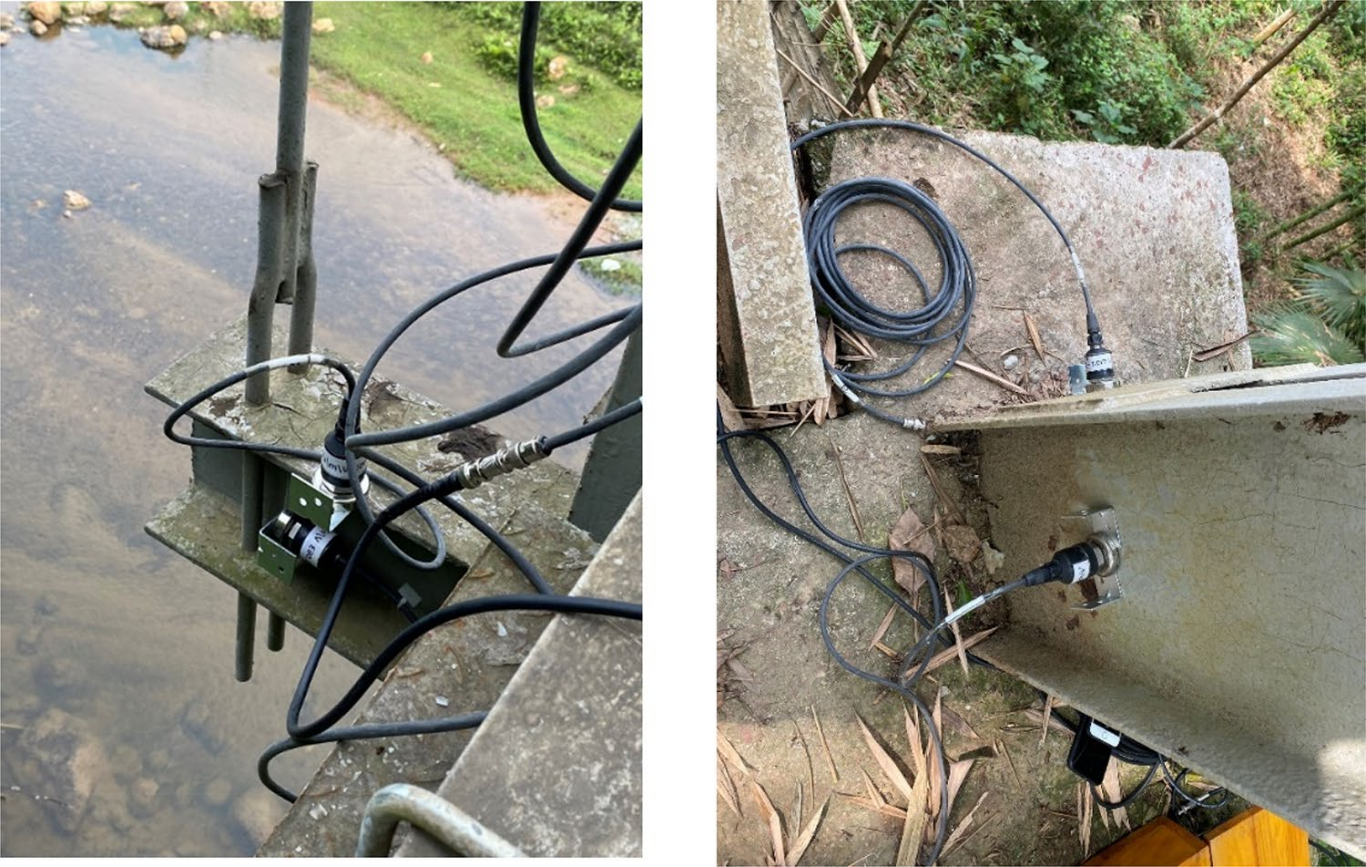
Installation of accelerometers on some of the vibration-sensitive points along the transversal beam (left) and tower (right).

The vibration measurement of Na Xa bridge was carried out from 19/04/2022 to 21/04/2022 with the aim of identifying the dynamic characteristics of the bridge after retrofitting in 2019, including natural frequencies and mode shapes. The data acquisition system consists of high-sensitivity accelerometers (PCB-393B12-sensitivity range: 965–1083 mV/g) and a data acquisition platform 08‑slot CompactDAQ chassis (cDAQ‑9178) connected to eight NI-9234 modules. The accelerometers were employed at vibration-sensitive points along the bridge (Fig. 5) to collect the dynamic response signals. The main sources of excitation considered were ambient (wind load) and pedestrian load. The vibration measurement was conducted in 20 min to collect vibration data from the whole bridge.

#### Modal analysis

**Figure 6:**
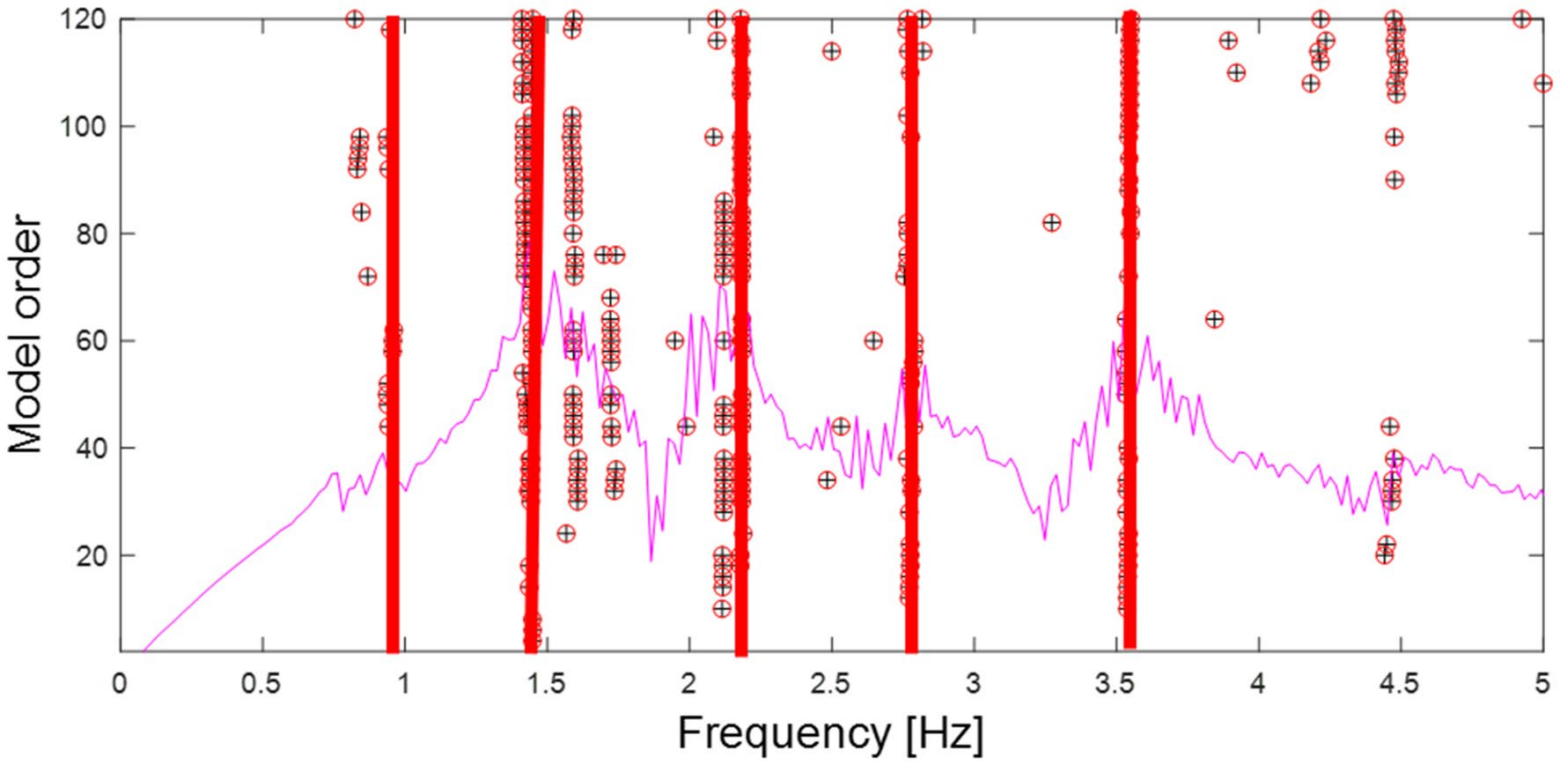
Stabilization diagram with the frequency interval between 0 and 5 Hz.

For the system identification and modal analysis of the measured data, MACEC toolbox 3.3 is used^36^. Fast Fourier Transform (FFT) was used to convert the measured time-series data to the frequency domain. The output-only data have been processed using OMA algorithms: covariance-based data-driven stochastic subspace identification (SSI-COV)^37^. The measured signals have been pre-processed with a concatenation of 20 min per measured setup; Fourth-order Butterworth filtering is used to filter the signal with a lowpass frequency of 5 Hz and a high pass frequency of 0 Hz to remove all phase distortion and subsequently present the frequencies range by a decimation factor of 160. To optimize the computational cost, the number of chosen block rows of the Hankel matrix is chosen as 120, and the model order range is deduced from 2 to 120 in step of 2. The stabilization diagram is obtained as shown in Fig. 6.

It is noteworthy that the presence of uncertainties during the measurement, especially from the environmental conditions, has caused many noises that can be encountered during the signal processing, which in turn causes difficulties in locating the exact natural frequencies of the structure from the stabilization diagram. In the end, five mode shapes are identified from the measurement data, which are correlated to the result from the initial FE model, as given in Table 1.

**Table 1:** Identified vibration modes from measurement data.

Mode number	Measured natural frequency (Hz)	Initial FE model natural frequency (Hz)	Mode shape type
1	0.90	0.66	Vertical bending
2	1.42	1.48	Vertical bending
3	2.15	2.09	Torsion
4	2.77	2.87	Torsion
5	3.51	3.91	Vertical bending

#### Validation of the FE model

From Table 1, we can see that although there exist correlations between the values of the measured natural frequencies of the bridge and the values of the initial FE model, the correlated errors are still significant, which can hinder the correctness of the FE model. To improve the FE model, model updating is conducted to obtain a baseline model of the bridge, which will be used to validate the damage detection procedure in the next section. It is achieved by identifying the different uncertainties of the bridge, namely the Young’s modulus of different bridge components. The result of the model updating is shown in Table 2.

**Table 2:** Comparison of natural frequencies before and after model updating.

Mode number	Measured natural frequency (Hz)	Initial FE model natural frequency (Hz)	Updated FE model natural frequency (Hz)	Difference between the initial FE model and measurement (%)	Difference between updated FE model and measurement (%)
1	0.90	0.66	0.85	26.7	5.51
2	1.42	1.48	1.47	4.05	3.62
3	2.15	2.09	2.15	2.79	0.16
4	2.77	2.87	2.75	3.61	0.74
5	3.51	3.91	3.52	11.40	0.24

From Tables 2 and 3, we can see that the natural frequencies of the updated FE model are closer to the actual values obtained from the measured data. The % differences between the natural frequencies of the model and the actual bridge are within the acceptable range. Therefore, the updated model will be used to validate the damage detection method using HBGA in the next section of the paper.

**Table 3:** Updating parameters for the FE model updating.

Parameter number	Component	Lower boundary (Pa)	Upper boundary (Pa)	Optimized value (Pa)
E1	Main beam	1.85 × × 10^11^	2.30 × 10^11^	1.85 × 10^11^
E2	Crossbeam	1.85 × 10^11^	2.30 × 10^11^	2.30 × 10^11^
E3	Main cable	1.85 × 10^11^	2.30 × 10^11^	2.05 × 10^11^
E4	Suspended cable	1.85 × 10^11^	2.30 × 10^11^	2.30 × 10^11^
E5	Bridge tower	1.85 × 10^11^	2.30 × 10^11^	2.30 × 10^11^
E6	Upper bracing	1.85 × 10^11^	2.30 × 10^11^	1.86 × 10^11^
E7	Crossbar on the deck	1.85 × 10^11^	2.30 × 10^11^	2.29 × 10^11^
E8	Lower bracing	1.85 × 10^11^	2.30 × 10^11^	2.23 × 10^11^
E9	Reinforced cable under the deck	1.85 × 10^11^	2.30 × 10^11^	1.85 × 10^11^
E10	U-shaped component connecting the main beam and the cross beam	1.85 × 10^11^	2.30 × 10^11^	2.30 × 10^11^

### Results and discussion of damage detection of Na Xa bridge using HBGA

**Figure 7:**
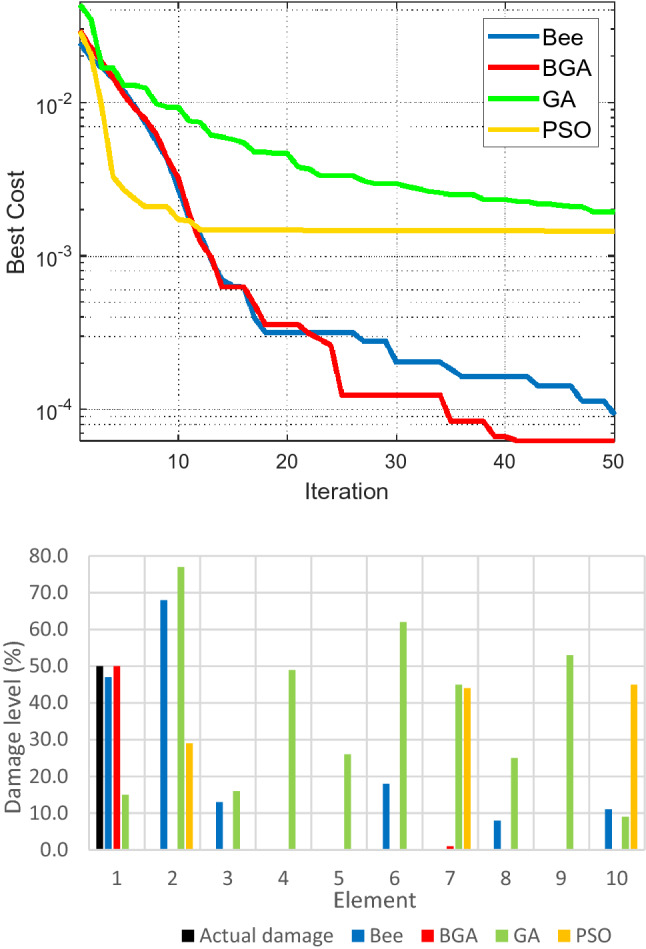
Convergence of fitness function (upper) and the location, quantification of damage (lower) for the simple damage case.

**Figure 8:**
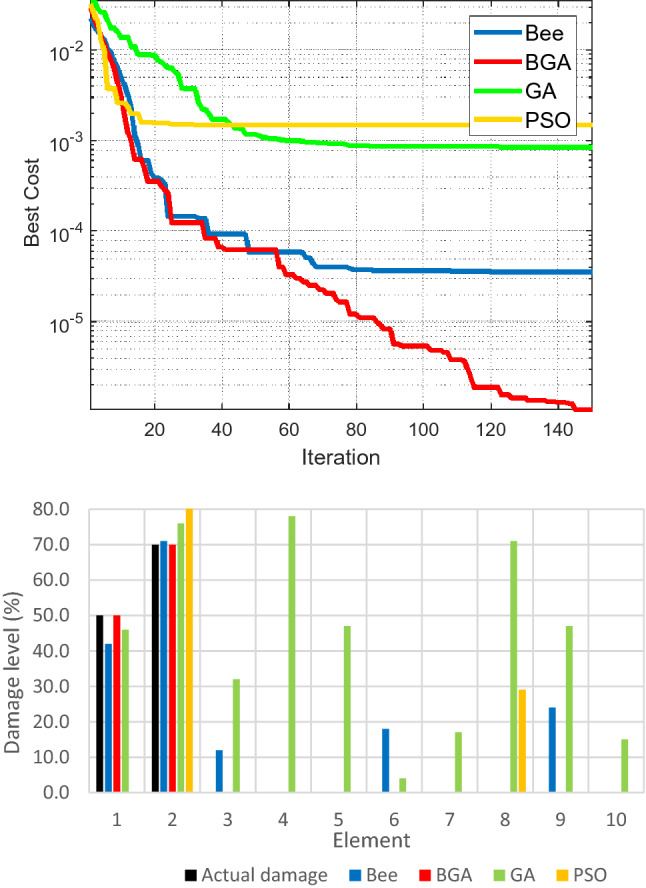
Convergence of fitness function (upper) and the location, quantification of damage (lower) for the multiple damage case.

To validate the damage detection capability of the proposed algorithm on Na Xa bridge, two damage scenarios are generated based on the FE model of the bridge after retrofitting. In the first case, single damage is simulated by reducing Young's modulus of one element at the main bridge beam to 50%. In the second case, multiple damages are generated by reducing Young's modulus of two beam elements at the girder beam to 50% and 70%, respectively. The objective function is calculated as follows:

27 $$\begin{array}{c}{\text{Objective\, Function}}=\sum_{i=1}^{5} {\left({f}_{i}-\widetilde{{f}_{i}}\right)}^{2}/{\widetilde{f}}_{i}^{2}\\ \end{array}$$

where ${f}_{i},$ ${\widetilde{f}}_{i}$ are the natural frequencies of the analytical and experimental results, respectively, for the first five vibration modes of the structure. HBGA is employed to search for the best value of the objective function. The local search is performed by BA to search for the area, which contains the best possible solution, while GA is employed to avoid local minima and identify the optimal solutions to the problem, which are the location and the corresponding severity of the damaged beam elements within the suspension footbridge structure. To evaluate the effectiveness of the proposed method, they are compared with the original BA, GA, and Particle Swarm Optimization (PSO), which are among the most widely used OAs due to their effectiveness and simplicity. For the two damage cases, each run consists of a population size of 50 to 500 and 50 to 150 iterations. The results of the two cases are shown in Figs. 7 and 8.

Figures 7 and 8 show that for both single and multiple damage scenarios, HBGA has a better convergence rate than BA, GA, and PSO. For example, BA identifies false damages at elements 2, 3, 6, 8, and 10; GA detects damage incorrectly for all the considered elements; PSO gives wrong damage levels at elements 2, 7, and 10. For the multiple damage case, the same thing happens with false damages appearing at the incorrect locations in the case of BA, GA, and PSO: BA wrongly identifies damage at elements number 2, 3,6, and 9, respectively.; GA read that there exist damages in all the search elements, which is not the case considered; PSO is unable to identify the actual damage at element number 1, while it also falsely identifies damage at element number 9. From there, it can be deduced that the proposed HBGA outperforms the other considered algorithms (BA, GA, PSO) in damage identification and quantification of structure in both the single and multiple damage cases of Na Xa bridge.

In terms of computational cost, as shown in Table 4, HBGA also performs much better than BA, GA, and PSO for both single and multiple damage cases. Specifically, HBGA requires 4540 s to determine the best solution when single damage is generated in the structure, while BA, GA, and PSO cost 8029 s, 8145 s, and 8510 s, respectively. For multiple damage cases, the computational cost of HBGA is 4552 s, and for BA, GA and PSO it costs 7635 s, 7714 s, and 8525 s. It shows that HBGA is better than the other algorithms in detecting structural damages and much superior in reducing the computational cost required to solve the problem.

**Table 4:** Computational cost required for different damage cases.

Algorithm	CPU time (s)	
	Single damage case	Multiple damage case
HBGA	**4540**	**4552**
BA	8029	7635
GA	8145	7714
PSO	8510	8525

Significant values are in bold.

## Conclusion

Based on the analysis and result presented herein, the following conclusions can be drawn:

(1) The proposed HBGA, which combines both the strength of BA and GA, can identify damages generated in a baseline model of suspension footbridge with a high level of accuracy. (2) The accuracy of the proposed method is also proven to be much superior when compared with the other optimization algorithms (BA, GA, and PSO). (3) The efficiency of the method is further assured by the computational cost statistic results.

This paper proposes a novel adaptive hybrid metaheuristic optimization algorithm, namely HBGA, to solve the damage detection problem of suspension footbridges. The HBGA is able to fuse both the strengths of the classic Bee Algorithm and Genetic Algorithm into a powerful technique that can not only identify damages, but also quantify their severity accurately, as demonstrated in the chosen case study of Na Xa bridge. For the validation of its effectiveness, the proposed HBGA outperformed both its makers as well as the widely used PSO algorithm. It is noteworthy that the proposed method has plenty of room for improvement in future research. This method can also be extended to solve various optimization of other lifeline infrastructures such as dams, skyscrapers, etc.

## Data Availability

The datasets used and/or analysed during the current study available from the corresponding author on reasonable request.
